# Tirofiban-induced acute profound thrombocytopenia in endovascular therapy for acute ischemic stroke: a rare case report

**DOI:** 10.1186/s12883-025-04368-1

**Published:** 2025-08-27

**Authors:** Jiuling Liu, Melysze Deanne Oorloff, Adithya Nadella, Yun Yan, Ning Zhou

**Affiliations:** 1 Department of Neurology, Nanjing BenQ Medical Center, the Affiliated BenQ Hospital of Nanjing Medical University, Nanjing, Jiangsu Province China; 2 Nanjing Medical University, Nanjing, Jiangsu Province China

**Keywords:** Tirofiban, Thrombocytopenia, Endovascular

## Abstract

**Background:**

Studies have shown that tirofiban (a glycoprotein IIb/IIIa receptor antagonist) is effective in the endovascular treatment of cerebrovascular diseases. However, it has also been reported that tirofiban can cause thrombocytopenia. Here we report a rare case of tirofiban-induced acute profound thrombocytopenia in a patient undergoing endovascular therapy for acute ischemic stroke.

**Case report:**

A 39-year-old male patient was sent to the emergency department of our hospital due to a sudden onset of disturbance in consciousness, aphasia and right hemiparesis for 2 h. Computed tomography angiogram (CTA) revealed an occlusion of the left middle cerebral artery. He was directed into emergency embolectomy with stent implantation under digital subtraction angiography (DSA) guidance. Due to the extensive clot formation during surgery, a bolus of 20 ml tirofiban was slowly injected through the guiding catheter. Approximately two hours after the infusion, the patient developed acute profound thrombocytopenia.

**Conclusion:**

Tirofiban-induced thrombocytopenia after drug infusion may occur acutely and without any bleeding manifestations. There may be a clinical correlation between the loading dose of tirofiban and the occurrence of acute profound thrombocytopenia. Clinicians should maintain high caution for the potential crash of platelet levels when this drug is administered during procedures.

## Introduction

Tirofiban is an antithrombotic agent glycoprotein IIb/IIIa receptor antagonist, and is a drug widely used in patients receiving interventional therapy due to cerebrovascular diseases [1]. Studies have demonstrated that mechanical thrombectomy with solitaire AB stents, combined with tirofiban injection through a microcatheter, appears safe and effective in the endovascular treatment of ischemic stroke [2, 3]. Tirofiban-induced thrombocytopenia is a known but rare adverse effect [4]. Tirofiban has been extensively studied in clinical research involving patients with coronary artery syndrome and percutaneous coronary intervention (PCI) and is widely used in clinical practice in China. In the PRISM [5], RESTORE [6], and PRISM-PLUS [7]studies, the incidence rates of tirofiban-associated thrombocytopenia were 0.4%, 1.1%, and 1.9%, respectively. The above data indicates that although tirofiban carries a risk of inducing thrombocytopenia, its overall incidence rate remains at a relatively low level. An expanding body of evidence points to tirofiban changing the configuration of glycoprotein receptors on the platelets, producing a novel antigen that is then identified and eliminated from circulation as a result of an immune-mediated response [6]. Extremely few cases of profound thrombocytopenia (platelet count < 20 × 10⁹/L) have been described, with an incidence of just 0.2–0.5%. The adverse event typically occurs within the first 24 h of treatment and is often accompanied by bleeding complications. Platelet counts may return to normal within 1 to 6 days after discontinuation of the drug [6, 8].

### Case report

Herein, we report a case of acute profound thrombocytopenia induced by tirofiban in a patient who underwent emergency comprehensive endovascular treatment for occlusive infarction of the left middle cerebral artery. He was treated with tirofiban to inhibit platelet aggregation during and after surgery, and the platelet count abruptly plummeted to 2 × 10^9^/L within 2 h of administration.

**Fig. 1:**
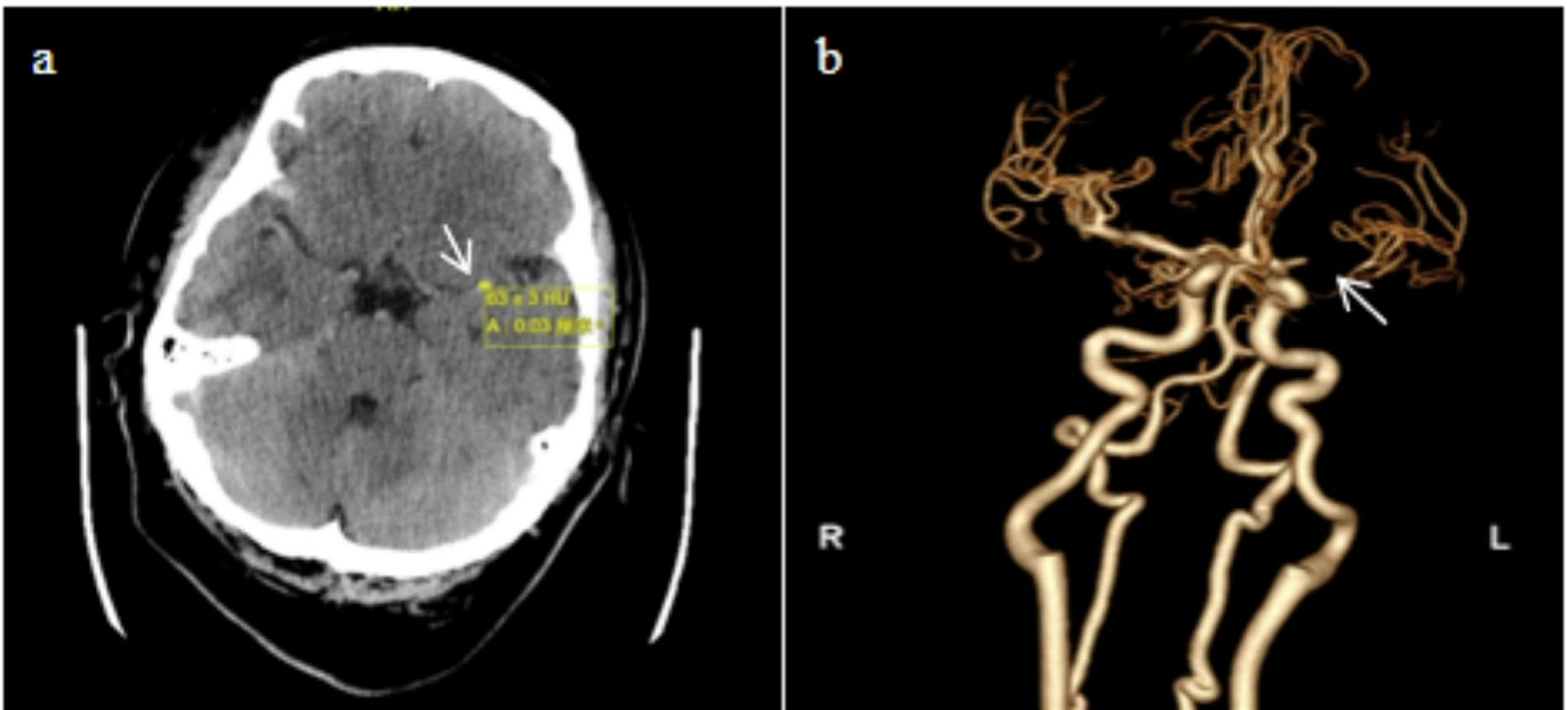
Preoperative imaging examination. **a**Brain computed tomography showed a high density of the left MCA. **b** Computed tomography angiography (CTA) showed a left M1 occlusion

A 39-year-old male patient was sent to the emergency department of our hospital due to a sudden onset of impaired consciousness, aphasia and right hemiparesis for 2 h. On neurologic examination, the patient had a Glasgow coma scale (GCS) score of 7, global aphasia, conjugate rightward gaze deviation and right hemiparesis. The National Institute of Health Stroke Scale (NIHSS) score was 22. Brain computed tomography (CT) showed a high-density sign of the left middle cerebral artery (MCA) (Fig. 1a). Computed tomography angiogram (CTA) revealed an occlusion of the left M1 MCA (Fig. 1b). The patient’s family did not agree to receive intravenous thrombolysis (IVT) but requested direct thrombectomy. Therefore, we urgently performed direct mechanical thrombectomy of the left MCA after obtaining consent from the family members.

**Fig. 2:**
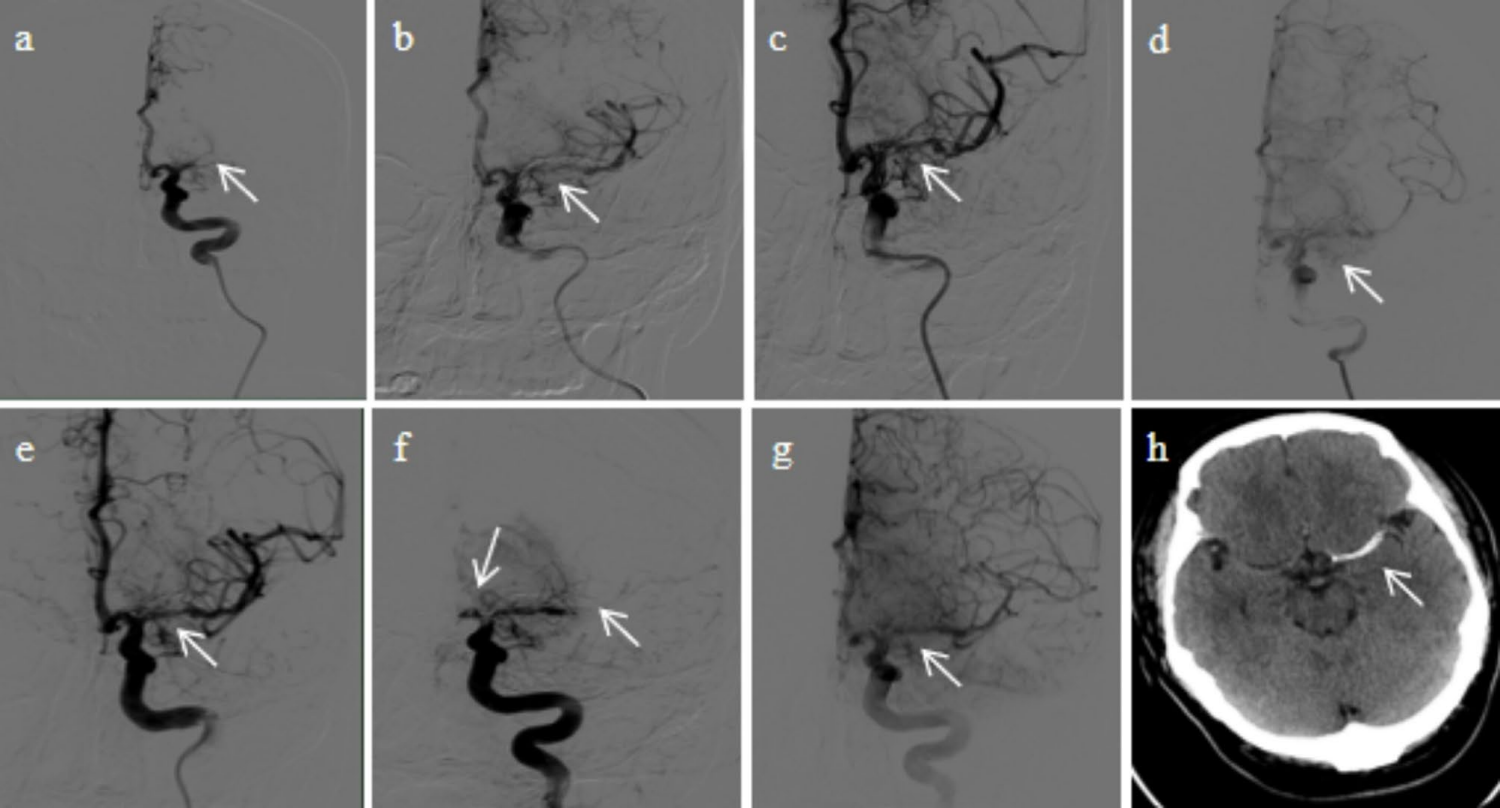
Intraoperative Images. **a** Left M1 occlusion. **b** A solitaire stent (4 mm×20 mm) was placed in the occluded segment and the left MCA M1 was recanalized. **c** Post-mechanical thrombectomy angiogram revealed the left MCA is unobstructed. **d** Repeated carotid angiography revealed the left MCA is hemodynamically unstable with mTICI grade 2a. **e** Recanalization of left MCA M1 segment after stent implantation. **f** The angiography showed mass thrombosis in the stent, ACA and left MCA occluded. **g** Angiography showed recanalization of the ACA and left MCA. **h** Post-procedure CT brain showed no hemorrhage and a stent in the M1 segment of the left MCA

An 8 F sheath was inserted into the right femoral artery of the patient, and cerebral angiography showed occlusion of the left MCA in the M1 segment (Fig. 2a). The 6 F guiding catheter tip was indwelled at the end of the petrous segment of the left internal carotid artery under the guidance of a super-slip guide. Using the Navien catheter, microguides were carefully placed in the M3 segment of the left MCA through the M1 occlusion segment of the left MCA. Subsequently, angiography confirmed the occlusion in the true lumen. A solitaire stent (4 mm×20 mm, EV3, California, USA) was placed in the occluded segment and the left MCA was recanalized (Fig. 2b). After 5 min of observation, the stent was removed and Navien catheter aspiration was performed (Fig. 2c). A large amount of thrombosis was removed. However, post-mechanical thrombectomy angiogram revealed hemodynamic instability with mTICI grade 2a (Fig. 2d). At this point, we decided to deploy a solitaire stent (4 mm×20 mm, EV3, California, USA) in the M1 occlusion segment of the left MCA, which immediately restored flow (Fig. 2e). After 15 min of observation, the angiography showed mass thrombosis in the stent and the anterior cerebral arteries (ACA) and left MCA were occluded (Fig. 2f). Since the clot burden was significant, a bolus of 20 ml (1 mg) tirofiban was slowly injected through the guiding catheter. After 20 min of observation, angiography showed recanalization of the ACA and left MCA with mTICI grade 3 (Fig. 2g), the procedure was completed successfully. A continuous infusion of tirofiban (0.1 ug/kg/min IV) was given by the peripheral vein after the operation, and the patient was transferred to the Intensive Care Unit (ICU) for further treatment. Throughout the procedure, peripheral venous heparinization was performed with a heparin dosage of 3000 IU.

**Fig. 3:**
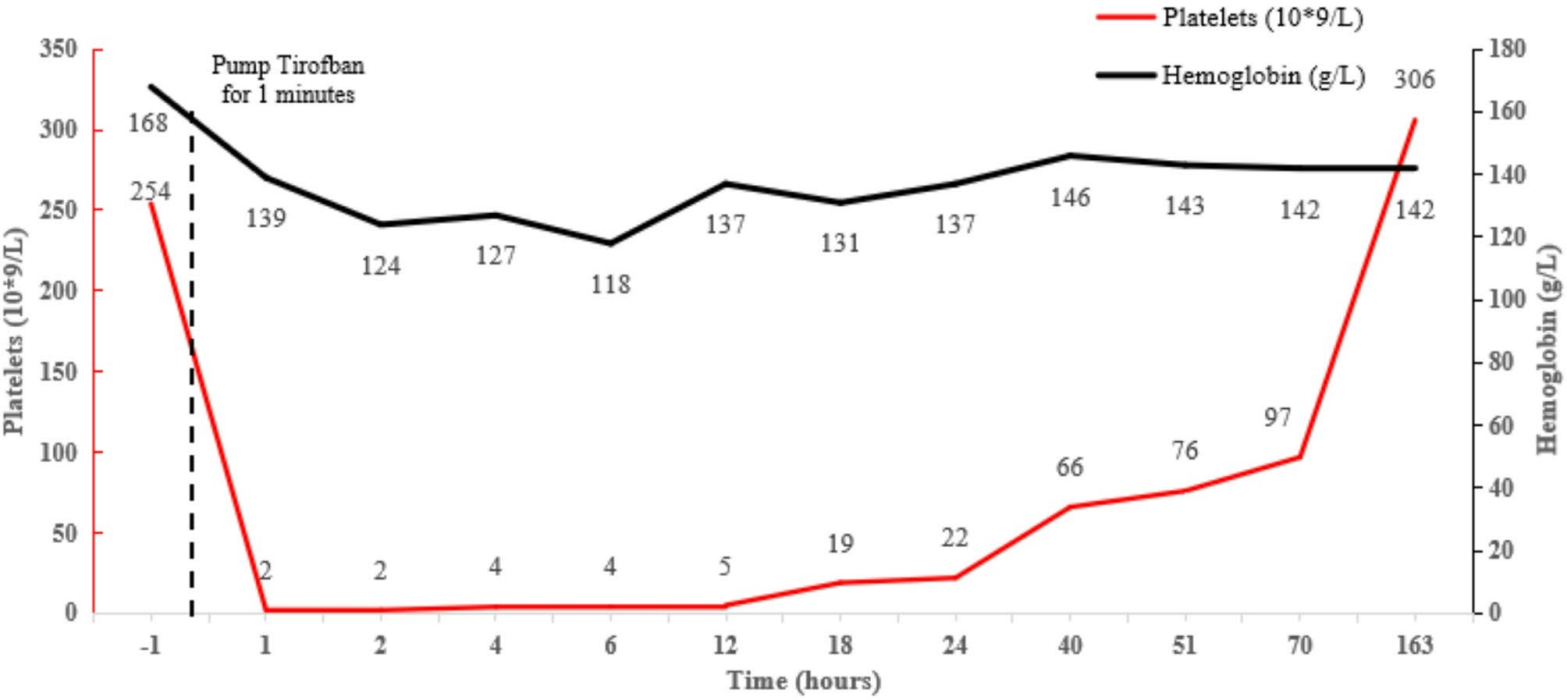
Platelet count and hemoglobin levels before and after tirofiban infusion

Before the surgery, the patient’s platelet count was within the normal range at 254 × 10^9^/L. However, after the surgery, a blood routine examination showed a significantly low platelet count of 2 × 10^9^/L. Fibrinogen, D-dimer, thrombin time (TT), and international normalized ratio (INR) of prothrombin were all within the normal range. The plasma prothrombin time (PT) was 10.1 s (normal range: 10.7 to 14.4 s), and the activated partial thromboplastin time (APTT) was 19 s (normal range: 23.5 to 35 s). Routine coagulation parameters showed no significant changes. The re-examination of blood platelet levels indicated similar results. Tirofiban was discontinued immediately, and the patient was given one unit platelet transfusion. Blood routine tests were repeated on postoperative day 1, and platelet count showed slight improvement. Figure 3 shows the platelet count over the course of admission. Furthermore, the patient showed no signs of bleeding or significant changes in the hemoglobin levels **(**Fig. 3**)**. Patient’s peripheral blood smear showed no signs of platelet clumping, ruling out pseudothrombocytopenia. A thromboelastography heparin test was subsequently performed, revealing no residual heparin. Following this, vascular ultrasound of the extremities showed progressive improvement, indicating smooth blood flow without thrombus formation. Meanwhile, the platelet membrane glycoprotein antibody GPIIb/IIIa tested positive, while enzyme linked immunosorbent assay (ELISA) test for antiheparin/PF4 antibodies was negative.

The patient was transferred to the neurology department of the hospital on postoperative day 3, and blood platelet levels were closely monitored. Platelet count improved to 66 × 10^9^/L, and Argatroban (continuous intravenous infusion at 60 mg/d for the first 48 h and then at 10 mg twice daily) was initiated to lower the risk of reinfarction or stent thrombosis. After the first dose of Argatroban, the blood tests showed that platelet levels continued to rise, and thereafter, Aspirin-enteric-coated tablets (100 mg/qd) were added to the treatment regimen. On postoperative day 6, Argatroban was stopped, and based on the Gene Test Report of antithrombotic drugs; Ticagrelor tablets were added to the treatment plan. Transcranial Doppler (TCD) and a cephalic CTA examination were used to monitor blood flow and showed an improvement in blood flow through the stent. Two weeks after the discontinuation of tirofiban, the patient’s platelet levels recovered to greater than the preoperative level.

## Discussion

Tirofiban acts on the final stage of platelet aggregation by selectively inhibiting GPIIb/IIIa receptors and can inhibit platelet aggregation instantly. In recent research and guidelines, an appropriate dose of tirofiban has been recommended as an important short-term antithrombotic agent for the intravascular treatment of acute ischemic stroke field [9, 10]. However, Tirofiban has been reported to be associated with thrombocytopenia. It’s crucial to rule out other causes of thrombocytopenia when a patient receiving Tirofiban, such as heparin, in order to confirm the diagnosis [11].

Four different kinds of acquired thrombocytopenia are commonly encountered during the perioperative stage of interventional surgery. They are pseudo-thrombocytopenia, heparin-induced thrombocytopenia, aspirin and adenosine diphosphate (ADP) receptor antagonist-induced thrombocytopenia, and glycoprotein IIb/IIIa receptor antagonist-induced thrombocytopenia (GIT) [12]. Pseudo-thrombocytopenia is a condition in which platelets are incorrectly counted as a result of clumping in blood samples that have been treated with ethylene diamine tetraacetic acid (EDTA) [11]. It is required to distinguish between pseudo-thrombocytopenia and true thrombocytopenia to avoid premature discontinuation of drugs. Repeated laboratory testing on our patient has ruled out this diagnosis. Due to the patient received 3000 IU of heparin during the operation. Heparin-induced thrombocytopenia was suspected in the differential diagnosis. Heparin-induced thrombocytopenia (HIT) is classified into two types: Type I and Type II. Type I HIT is a non-immune reaction that typically occurs within 1 to 2 days of heparin administration. It is characterized by a mild decrease in platelet count, generally fluctuating between 100 and 300 × 10⁹/L, and rarely falling below 100 × 10⁹/L. In this case, however, the platelet count dropped drastically from 254 × 10⁹/L to 2 × 10⁹/L within one hour, with no residual heparin detected in the blood sample. Given the presence of acute and severe thrombocytopenia, Type I HIT was effectively ruled out. Type II HIT is immune-mediated and usually manifests between 4 and 14 days after initial heparin exposure. It is associated with a significant decline in platelet count, and in this case, the possibility of Type II HIT could not be initially excluded. The 2012 US clinical guidelines recommend using a “4Ts” scoring system to assist in diagnosing suspected HIT patients. The 4Ts include thrombocytopenia, time to onset of platelet decline, thrombosis, and alternative causes of thrombocytopenia [13]. In our case, within 24 h of using Unfractionated Heparin (UFH), the platelet count decreased to a minimum of 2 × 10^9^/L with no previous history of heparin exposure and recurrent formation of thrombi during surgery. However, there was no prior heparin exposure, no evidence of thrombosis on Doppler ultrasound of the extremities, and no clinical symptoms indicative of thromboembolism. Based on these factors, the patient received a combined 4Ts score of 2, making HIT unlikely. Furthermore, Type II HIT is characterized by the production of heparin-PF4 antibodies, however ELISA test for antiheparin/PF4 antibodies was negative, further ruled out Type II HIT. Therefore, we concluded that thrombocytopenia was not due to HIT but was more likely caused by tirofiban and was withdrawn.

Additionally, it should be noted that aspirin and ADP receptor antagonist, such as clopidogrel-induced thrombocytopenia, is extremely rare [14]. ADP receptor blockers specifically inhibit platelet aggregation by preventing both the binding of ADP to its platelet receptors and the secondary activation of ADP-mediated activation of glycoprotein IIb/IIIa complexes [12]. This patient was not administered clopidogrel and aspirin prior to the platelet decline. One characteristic that sets GIT apart is its tendency to manifest within a 24-hour timeframe, and in some cases, within a range of 30 min to several hours following the administration of said agents [15]. The risk of bleeding is higher in this form of thrombocytopenia, and it can occasionally lead to severe complications along with serious bleeding. Platelet transfusion is recommended in patients who develop life-threatening- thrombocytopenia (platelet count < 10 × 10^9^/L) [16]

Although the etiology of this phenomenon remains unclear, it is proposed that drug-dependent antibodies which bind platelets in the presence of tirofiban is the cause of the rapid fall of platelet levels. Routine coagulation parameters are typically unaffected by tirofiban. Nevertheless, when the drug is stopped and cleared from circulation, thrombocytopenia is seen to subside. These antibodies are naturally occurring or induced by conformational changes to glycoprotein IIb/IIIa receptor [17]. If antibodies are naturally occurring, which we believe to be the case for our patient, thrombocytopenia is acute (onset within < 24 h) and severe but resolves quickly after discontinuing the causative drug.

While the typical dose range of tirofiban ranges from 0.25 mg to 0.5mg [18], our patient was given a tirofiban bolus of 20 ml (1 mg) due to the high clot burden during the procedure. This high dose might have had a significant effect on the drastic drop in the platelet count that ensued within a few hours of tirofiban administration. We believe that this case is unique because the thrombocytopenia was acute and profound (reported a very low count of 2 × 10^9^/L). There is reason to consider that this case may have occurred due to the high dose of tirofiban that was administered during the stenting procedure. However, more evidence is needed to confirm the effect of the loading dose of tirofiban on the occurrence of acute profound thrombocytopenia.

## Conclusion

This case highlights acute profound thrombocytopenia after a high dose of tirofiban during a cerebral stenting procedure. There may be a clinical correlation between the loading dose of tirofiban and the occurrence of acute profound thrombocytopenia. However, further research is needed to confirm this. Given the rapidity of the onset of thrombocytopenia and the lack of bleeding manifestations, our case emphasizes the need to closely monitor platelet counts after tirofiban administration to prevent life-threatening complications. 

## Data Availability

No datasets were generated or analysed during the current study.

## Abbreviations

CTA: Computed tomography angiography
DSA: Digital subtraction angiography
GCS: Glasgow Coma Scale
NIHSS: National Institute of Health Stroke Scale
CT: Computed tomography
IVT: Intravenous thrombolysis
ICU: Intensive Care Unit
HIT: Heparin-Induced thrombocytopenia
TCD: Transcranial Doppler
ADP: Adenosine diphosphate
GIT: Glycoprotein IIb/IIIa receptor antagonist-induced thrombocytopenia
UFH: Unfractionated Heparin
EDTA: Enzyme linked immunosorbent assay
